# Finding inhibitors for PCSK9 using computational methods

**DOI:** 10.1371/journal.pone.0255523

**Published:** 2021-08-05

**Authors:** Rida Zainab, Afshan Kaleem, Michał B. Ponczek, Roheena Abdullah, Mehwish Iqtedar, Daniel C. Hoessli

**Affiliations:** 1 Department of Biotechnology, Lahore College for Women University, Lahore, Pakistan; 2 Department of General Biochemistry, Faculty of Biology and Environmental Protection, University of Lodz, Lodz, Poland; 3 Panjwani Center for Molecular Medicine and Drug Research, International Center for Chemical and Biological Studies, University of Karachi, Karachi, Pakistan; Brooklyn College of the City University of New York, UNITED STATES

## Abstract

Proprotein convertase subtilisin/kexin type 9 (PCSK9) is one of the key targets for atherosclerosis drug development as its binding with low-density lipoprotein receptor leads to atherosclerosis. The protein-ligand interaction helps to understand the actual mechanism for the pharmacological action. This research aims to discover the best inhibitory candidates targeting PCSK9. To start with, reported ACE inhibitors were incorporated into pharmacophore designing using PharmaGist to produce pharmacophore models. Selected models were later screened against the ZINC database using ZINCPHARMER to define potential drug candidates that were docked with the target protein to understand their interactions. Molecular docking revealed the top 10 drug candidates against PCSK9, with binding energies ranging from -9.8 kcal·mol^-1^ to -8.2 kcal·mol^-1^, which were analyzed for their pharmacokinetic properties and oral bioavailability. Some compounds were identified as plant-derived compounds like (S)-canadine, hesperetin or labetalol (an antihypertensive drug). Molecular dynamics results showed that these substances formed stable protein-ligand complexes. (S)-canadine-PCSK9 complex was the most stable with the lowest RMSD. It was concluded that (S)-canadine may act as a potential inhibitor against atherosclerosis for the development of new PCSK9 inhibitory drugs in future *in vitro* research.

## Introduction

Familial hypercholesterolemia (FH) is a known inheritable disease. It can lead to elevation of low-density lipoprotein (LDL) cholesterol levels in the blood. It increases the risk of coronary artery disease (CAD) as it can lead to an increase in LDL level of more than 5 mmol/L (>190 mg/dL) [1]. The risk of atherosclerotic cardiovascular disease (ASCVD) increases in patients, who already have mutations in FH genes such as *LDLR*, *PCSK9* or *ApoB*, when compared to patients without any mutation, but with similar cholesterol levels [2]. Mutated FH related genes may lead to an increased level of LDL cholesterol (LDL-C) [3] leading to malfunction of the vascular endothelium.

ASCVD, including CAD, is a well-known disease in which endothelial dysfunction is observed. Endothelial dysfunction is the first stage that leads to atherogenesis. Hypercholesterolemia is known as one of the most frequent causes of endothelial dysfunction, inducing constriction of large blood vessels in the heart [4]. Cholesterol itself is needed for many physiological processes. The cell membrane is a major subcellular organelle requiring high amounts of cholesterol. Furthermore, cholesterol plays an important role in the synthesis of bile acid and steroid hormones. Increase in the amount of cholesterol, specifically under oxidizing conditions, may lead to atherosclerosis resulting in carotid, peripheral vessels’ and coronary heart disease [5].

Atherosclerosis is one of the main causes of stroke and heart attack. Arterial plaques may be formed as a result of cholesterol accumulation leading to atherosclerosis. Atherosclerotic cardiovascular events are directly linked to LDL-C and apolipoprotein B-100 (apoB-100) elevation. As retention of apoB in the walls of arteries can initiate inflammation leading to atherosclerosis development, a decrease in LDL-C levels may lead to a reduction of the risk of cardiovascular events [6]. Due to inflammatory changes in the walls of arteries, LDL-C deposits can lead to plaque formation, and decrease the blood flow to various organs. The proprotein convertase subtilisin/kexin type 9 (PCSK9) binds to the LDL receptor (LDLR), and leads to the degradation of liposomes enhancing atherosclerosis. Therefore, inhibition of PCSK9 is essential for the prevention of cardiovascular disease risk [7].

PCSK9 is a subtilisin component family of proteins or member of the kexin-like proconvertases family. The primary structure of PCSK9 consists of 692 amino acids and expression of this protein is seen primarily in the kidney, liver and intestine. It contains a signal peptide of 30 amino acids followed by three domains. The first domain is known as a prodomain (residues 31 to 152), the second is a catalytic domain (residues 153 to 451) and the third and last is the *C*-terminal domain (residues 452 to 692) [8]. The endoplasmic reticulum (ER) is the subcellular location for PCSK9 synthesis and its nascent 73 kDa peptide is a zymogen. Several modifications are observed in this zymogen till it reaches the cell surface. Autocatalytic cleavage activity occurs in between amino acids 152 and 153. In the secretory pathway, the prodomain present in the *N*-terminal remains tightly associated with the catalytic domain [9].

PCSK9 is a type of protease that can degrade LDLR following interaction with the domain of LDLR present extracellularly. PCSK9 has a subtilisin-like catalytic domain that can bind to LDLR EGF-A (epidermal growth factor-like repeats) domain. This domain contains Asp-374, which is known to be mutated to Tyr and enhances the affinity of PCSK9 for LDLR [9]. The binding of LDL to LDLR is important to decrease cholesterol accumulation and the risk of coronary artery disease. The binding of PCSK9 to LDLR prevents binding of LDL to LDLR, and promotes accumulation of cholesterol. Therefore, it is essential to inhibit PCSK9 and its association with LDLR to prevent CAD [10]. Therapies targeted against PCSK9 are based on two main strategies, biological therapies with monoclonal antibodies and small-molecule pharmacotherapies. Unfortunately, progress in discovering small-molecule pharmacotherapy for PCSK9 inhibition is difficult, because the PCSK9 molecular structure needed for small molecule binding to reduce this protein activity, has not been localized [11]. To find new binding sites for the inhibition of a protein, computational techniques based on bioinformatics and cheminformatics may be helpful. Bioinformatics and cheminformatics deal with creating and manipulating databases and statistical algorithms to solve biological and chemical data management and analysis. Their tools can be used to identify and analyze drug targets, macromolecular biological structures and active sites. These tools can also identify a candidate drug molecule for a particular drug target and calculate their drug-likeness through docking and ADMET studies [12].

Drug candidates are ranked based on their binding affinities and, if required, their binding characteristics can be optimized. The concept of drug discovery based on the computational procedure is known as structure-based drug design, so *in-silico* methods can represent a wide and promising approach towards the discovery of a new target protein with the prediction of its biological activity. Thanks to the increasing computing power of computers, molecular dynamics has now become a more accessible computational technique with which it is possible to simulate the behavior of protein-ligand complexes and drug effects on biological macromolecules that can be calculated over time at the atomic level [13].

In this *in-silico* study known anti-atherosclerosis drugs were used, namely Captopril, Zofenopril, Enalapril, Ramipril, Quinapril, Perindopril, Lisinopril, Benazepril, Fosinopril, Cilazapril, Moexipril, Trandolapril, Allicin and Teprotide [14]. Applying computational methods like pharmacophore designing to known drugs, novel small inhibitors were found for PCSK9. To study the interactions between the PCSK9 target protein and computationally identified novel drugs, molecular docking was performed. This work is important for the development of new drugs against PCSK9 that could be administrated to treat heart diseases and control the rising problems related to high cholesterol for atherosclerosis prevention and treatment.

## Materials and methods

### 3D structure of the target protein (PCSK9)

The 3D structure of the target protein was retrieved from the PDB database (https://www.rcsb.org/search) [15]. The target site is where the interaction between PCSK9 and the EGF-A domain of LDLR occurs. This interaction involves the prodomain and *C*-terminal domain of PCSK9 [16] and elevates plasma LDL-C levels causing predisposition for CVD. After analyzing all the available structures containing the domains having an active site in the PDB database, PDB ID 6U26 [17] was finalized based on its high resolution (1.59 Å).

### PCSK9 active site identification

**Fig 1:**
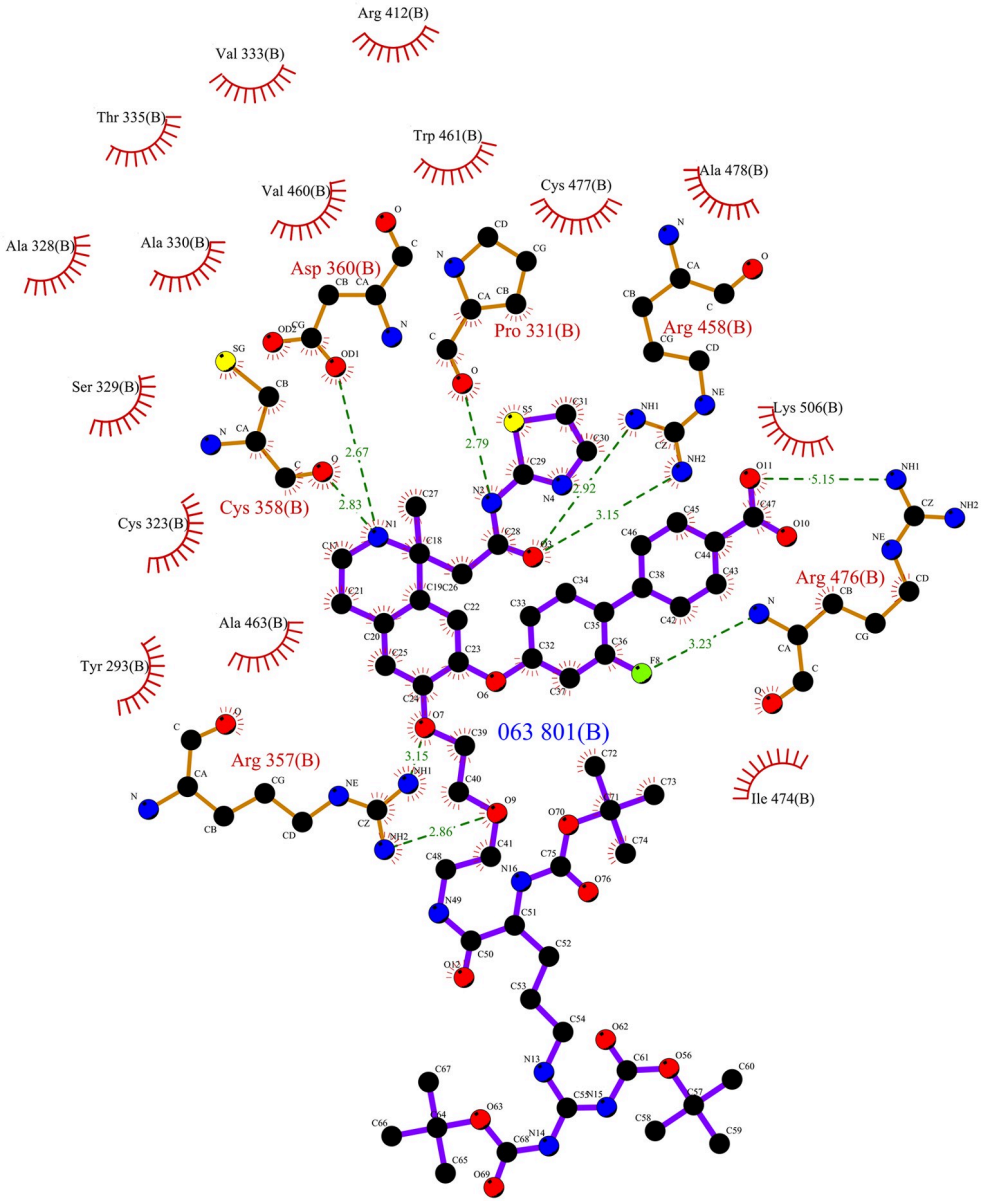
LIGPLOT for 6U26 showing the active site pocket from PDBsum [71].

The active site of PCSK9 (PDB ID 6U26) was located between the unusual allosteric pocket and the catalytic and *C*-terminal domain [17]. This pocket is represented by utilizing the LIGPLOT (Fig 1). LIGPLOT gives information about hydrogen bond interactions and hydrophobic contacts [18].

### Target protein and ligands’ preparation

The PDB ID 6U26 (target protein) was cleaned to remove heteroatoms, polar hydrogen, and Kollmann charges were added using Autodock tools [19]. The 3D structure of the target protein (PCSK9) was prepared by converting it to PDBQT format. For the preparation of the ligands, angiotensin-converting enzyme inhibitors (ACEi) showing activity against atherosclerosis, were identified (S1 Table). Their 2D structures were obtained from the PubChem database [20] using their respective PubChem IDs and energy minimized using Marvin Suite of ChemAxon (https://chemaxon.com/products/marvin). The energy minimized structures were converted to PDBQT format using Raccoon software [19].

### Molecular docking of known ACEi with PSCK9

The energy minimized structures (PDBQT format) were docked with the target protein using Autodock Vina [21]. The average value was found for binding energy and all the known molecules (Moexipril, Trandolapril, Allicin, Teprotide, Zofenopril, Ramipril and Quinapril) above the average value were chosen for pharmacophore designing. Binding energy, interactions (in the active site) and the structural comparison were the parameters used to finalize the inhibitors (S1 Table).

### Pharmacophore designing/modeling

Pharmacophore designing was carried out with PharmaGist [22] and the selection of pharmacophore was done based on the high score and maximum molecules aligned in the pharmacophore design. Pharmacophore is a 3-Dimensional setting of various features (hydrogen bond donor, hydrogen bond acceptor, anion/cation, aromatic ring, hydrophobic group), which aim for binding and are essential for a ligand to interact with a specific target [23].

### Virtual screening for the pharmacophore

Virtual screening is one of the standard steps for drug discovery before wet-lab experiments. This process involves the estimation of the binding affinity of the drug candidate towards a target protein. Virtual screening is also used to determine possible binding modes of the drug candidate and other drug-like small molecules against the target protein during the interaction. Most prominent drug candidates showing promising binding affinity towards target protein can be screened out using the High-Performance Computing (HPC) infrastructure tools [24]. Through the Virtual screening process, different bioactive molecules can be identified that can interact with the target protein [25]. Virtual screening for the pharmacophore was done using ZINCPHARMER [26] against the ZINC drug database and a total of 1033 hits/molecules were found.

### Molecular docking for final screened molecules

For docking the final set of 1033 screened molecules (using the designed pharmacophore) retrieved structures in SDF format (all molecules in one file) were used. These structures were separated and energy minimized using python script. The obtained structures were further converted to PDBQT format for Vina docking with the target protein. Docking of all the compounds was done using Autodock Vina [21] and the top 10 results (based on their interactions and binding energies) were analyzed using the Autodock Tools [19]. The grid and their x, y and z coordinates used in the docking were 40.516000, 24.375625 and 25.098375 respectively with grid dimensions of 25 Å each. The total number of modes were 20. The top 10 ZINC molecules, picked as final drug candidates, are given in S2 Table.

### Pharmacokinetic properties of the final drug candidates

Simplified molecular-input line-entry system (SMILES) for top ten compounds screened by Autodock Vina virtual screening, were converted by OpenBabel (http://openbabel.org/wiki/Main_Page) [27] from their PDBQT coordinates and the obtained SMILES codes were examined on ZINC (http://zinc.docking.org/substances/home/) [28], Molinspiration Cheminformatics free web services (http://www.molinspiration.com/), the Pubchem website, SWISSADME [29] and admetSAR [30]. The oral bioavailability of all the drugs was calculated using SWISSADME (S3 Table). The bioactivity prediction and drug-likeness from Lipinski rule of five were evaluated by the calculation of molecular properties and the bioactivity score by Predict Bioactivity tool available on (http://www.molinspiration.com/cgi-bin/properties). The LE and LELP parameters were calculated based on the results obtained by Molinspiration and molecular docking, where LE is Ligand Efficiency calculated from equations: LE=-RTlnKd=-ΔG0HA and LELP=miLogPLE (S4 Table).

### Molecular dynamics of protein-ligand complexes

Molecular dynamics of protein-ligand complexes were prepared and calculated in GROMACS [31], version 2018.4 on a personal computer with 6 core Intel(R) Core (TM) i5-8400 CPU @ 2.80GHz processor and GeForce GTX 1060 6GB NVIDIA graphics card. The GROMACS source code was downloaded from http://www.gromacs.org/ website and compiled in Microsoft Visual Studio Community MSVC 19.16.27025.1 with OpenCL version 1.2 from NVIDIA GPU Computing Toolkit CUDA v10.0 for Windows 10.

The PDBQT files of protein-ligand complexes, obtained as the result of virtual screening in Autodock Vina, were the starting source of coordinates, both for PSCK9 and screened hypothetical inhibitors. The coordinates in PDBQT format were converted using Open Babel into PDB format for protein and into MOL2 format for ligands. The obtained PDBQT file corresponding to the structure, 6U26, was next remodeled in SWISS-MODEL [32] with the whole human PCSK9 amino acid sequence (UniProt: Q8NBP7) [33, 34] to refill the loops missing in the original 3D structure. New PDB coordinates were downloaded and used to produce GRO protein topology by gmx pdb2gmx with CHARMM36 all-atom force field (July 2020). MOL2 files were supplemented with all the missing hydrogen atoms in the Avogadro software (http://avogadro.cc/) [35]. Updated MOL2 files were inspected visually and edited for any errors. The bonds in the coordinate files were segregated in ascending order using perl sort_mol2_bonds.pl script written by Justin A. Lemkul, and distributed by the author under the GPL-3.0 license.

Such processed MOL2 files were uploaded to generate ligand topologies on SwissParam (https://www.swissparam.ch/) to get ITP and PDB files. For each ligand, the obtained PDB file was converted to GRO by gmx editconf and the ITP file was included properly into corresponding topol.top file. The ligand GRO files were merged with protein GRO files in text editor and the final number of all the atoms was updated accordingly as the sum of the protein atoms and the given ligand atoms. The next step included defining the unit cell and filling it with water. Dodecahedron cell was created by gmx editconf from protein-ligand GRO file and proper topol.top file. Sodium and chloride ions were added to obtain 0.155 M concentration with a neutral charge and energy minimization was performed. The ligands and protein were restrained independently by generating position restraint topologies and two-phase 100 ps, 310 K and pressure 1 bar equilibrations were performed to generate at first NVT and next NPT outputs. Final molecular dynamics production was set for 10 ns of simulation in temperature 310 K and pressure 1 bar, and for one protein-ligand complex, a single simulation of a given protein-ligand complex lasted approximately 24 hours on the previously described computer equipment. The same temperature and pressure conditions were used for energy minimization and equilibration. Results were viewed in Visual Molecular Dynamics (VMD) (http://www.ks.uiuc.edu/Research/vmd/) [36] and the root-mean-square deviation (RMSD) was calculated by GROMACS gmx rms.

## Results

### 3D structure of the target protein (PCSK9) and its active site

**Fig 2:**
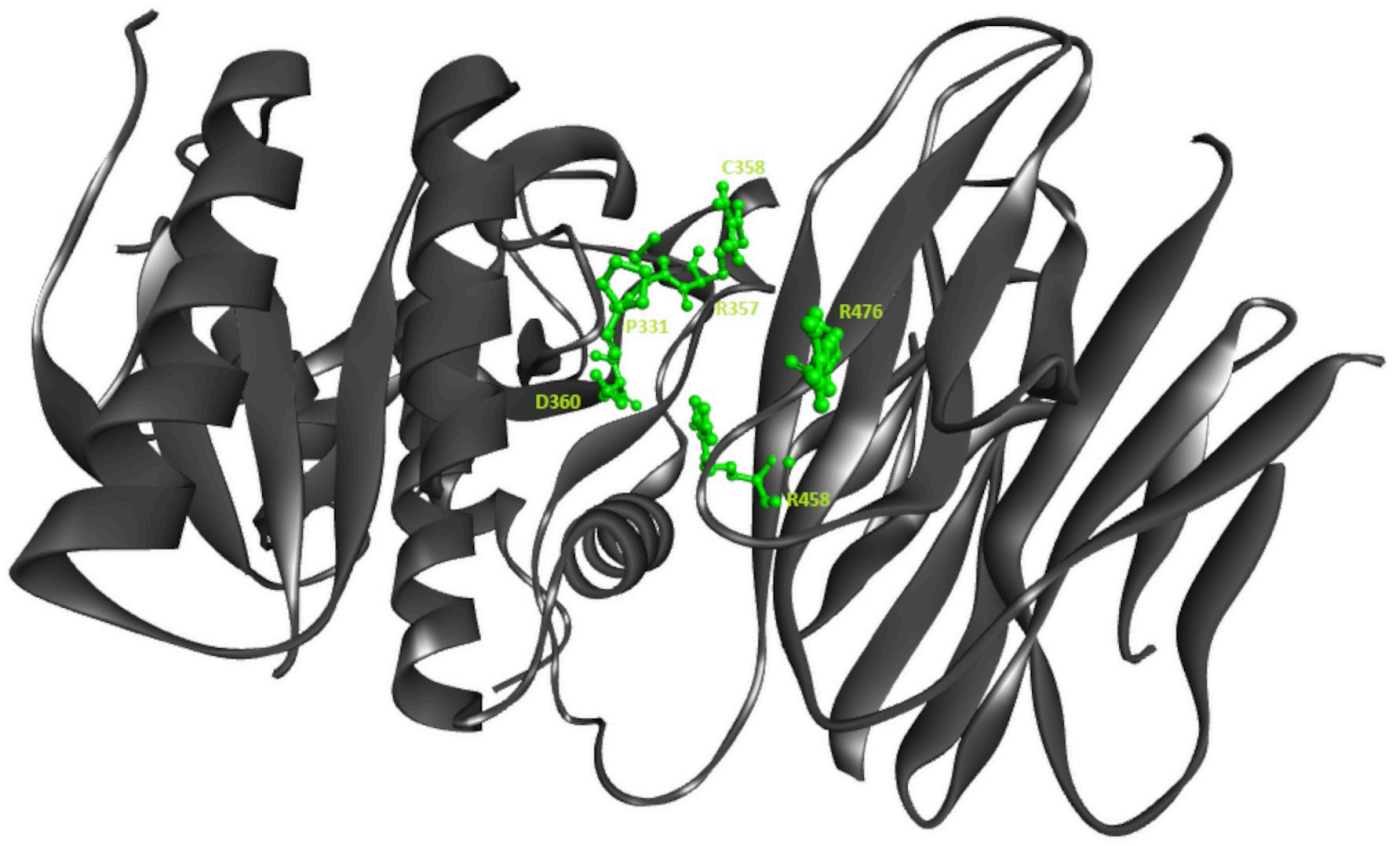
Image showing the active site pocket residues in ball and stick representation.

The LIGPLOT for the structure (PDB ID 6U26) showing the residues involved in the interaction is presented in Fig 1. The hydrogen bond interaction is represented with dashed lines between the ligand and amino acids of the protein. The hydrophobic interactions are represented by an arc with spokes around the interacting amino acids and radiating towards a portion of the ligand with which it is interacting. The interacting amino acids are D360, C358, R357, R476, R458 and P331. Fig 2 shows this interaction in contrast to distinguish between the active site residues from the entire structure using discovery studio.

### Selection of known inhibitors for pharmacophore designing using molecular docking studies

The literature was surveyed to find out the best structure for PCSK9 and its active site identified along with the known ACE inhibitors like Captopril, Zofenopril, Enalapril, Ramipril, Quinapril, Perindopril, Lisinopril, Benazepril, Fosinopril, Cilazapril, Moexipril, Trandolapril, Allicin and Teprotide (S1 Table). Docking was performed of all the known ACEi with the PCSK9 target protein and the top results were analyzed based on the computed binding energies and interactions in the active site.

### Pharmacophore based screened molecules

For this work, pharmacophores were used as a drug discovery approach to define novel inhibitors for PCSK9 using the selected known ACE inhibitors (Moexipril, Trandolapril, Allicin, Teprotide, Zofenopril, Ramipril and Quinapril, given in S1 Table). Pharmacophore models, generated by PharmaGist, were analyzed and the top pharmacophore was selected based on the high score of 18.102 and 5 features (1 hydrophobic, 1 negative ion and 3 hydrogen bond acceptors). Screening of the pharmacophore was done against the entire ZINC drug database in ZINCPHARMER and a total of 1033 hits/molecules were found.

### Molecular docking for finding top 10 molecules as best drug candidates

**Fig 3:**
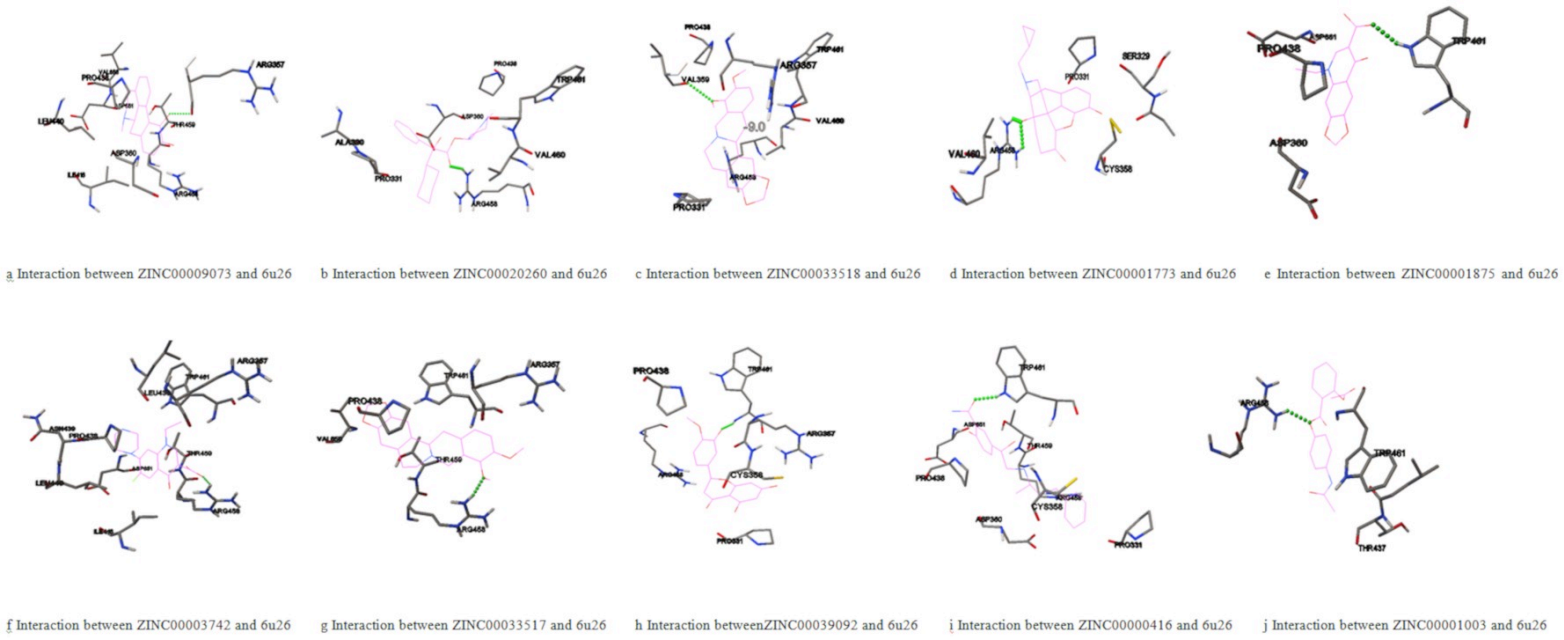
(a-j) Stick representation showing interactions between the top 10 molecules and target protein (PDB ID– 6U26).

After analyzing the molecular docking complexes of all the 1033 screened molecules with the target protein, their binding energies and interactions were recorded. Based on the lower binding energies as computed by AutoDock and interaction(s) in the active site as analyzed, the docking results were ranked to describe the top 10 ZINC molecules (S2 Table and Fig 3a–3j). Lower binding energies indicate higher binding affinity and stable interaction(s); therefore, the top molecules could give the predicted activity with the target protein (PCSK9) more efficiently and represent better options as drug candidates.

### Top 10 final drug candidates

The top 10 candidates were apomorphine, oxyphencyclimine, (S)-canadine, naltrexone, oxolinic acid, norfloxacin, (R)-canadine, hesperetin, labetalol and benorilate. S4 Table represents their PubChem CIDs, names, chemical formula, calculated properties and drug-likeness from the Lipinski rule of five. The first compound with the lowest docking free energy change is apomorphine (ZINC00009073) and according to PubChem, it is known as a non-selective dopamine agonist used in the treatment of Parkinson’s disease. The next one (ZINC00020260) is oxyphencyclimine–a muscarinic receptor antagonist given orally to treat peptic ulcer diseases and gastrointestinal spasms. The third (ZINC00033518) is (S)-canadine, also known as (S)-tetrahydroberberine and xanthopuccine. It is a benzylisoquinoline alkaloid (BIA), of the protoberberine [37] structural subgroup, and is present in many plants from the family *Papaveraceae* and genus Corydalis. The next compound (ZINC00001773) is naltrexone, a drug used primarily to manage alcohol or opioid dependence. ZINC00001875 is oxolinic acid, which is a synthetic antibiotic used in veterinary medicine. ZINC00003742 is Norfloxacin, also an antibiotic, with broad-spectrum antibacterial activity against most gram-negative and gram-positive bacteria. ZINC00033517 is (R)-canadine (other name tetrahydroberberine)–enantiomer of (S)-canadine. ZINC00039092 is hesperetin, a plant secondary metabolite described as an antioxidant and an antineoplastic agent. ZINC00000416 is 2-hydroxy-5-[(1S)-1-hydroxy-2-[[(2R)-4-phenylbutan-2-yl]amino]ethyl] benzamide, also called labetalol, a drug used to treat high blood pressure. It is a third-generation selective alpha-1-adrenergic antagonist and non-selective beta-adrenergic antagonist with vasodilatory and antihypertensive properties. The last compound (ZINC00001003) is benorilate–an ester-linked codrug of aspirin with paracetamol. It is used as an anti-inflammatory and antipyretic drug [38].

### Pharmacokinetic properties and the oral bioavailability of the final drug candidates

Computational structural analysis, drug-likeliness, ADME properties, oral bioavailability and toxicity profiling was done using Molinspiration, KNIME, SWISSADME and admetSAR (S3 and S4 Tables). The top molecules were back searched against the ZINC database, Molinspiration and PubChem using SMILES codes and all of them were recognized as known compounds, some of them of plant origin like (S)-canadine and hesperetin. Another compound, labetalol, was identified as an antihypertensive drug (S4 Table). Some of the chemical compounds containing nitrogen atoms may exist in more than one form depending on the degree of nitrogen protonation. These compounds have more than one PubChem code, alternative SMILES, but are referred in this database as “parent compounds”. Therefore, it can be considered that they are the same compounds that take different forms depending on the pH of the solution.

**Fig 4:**
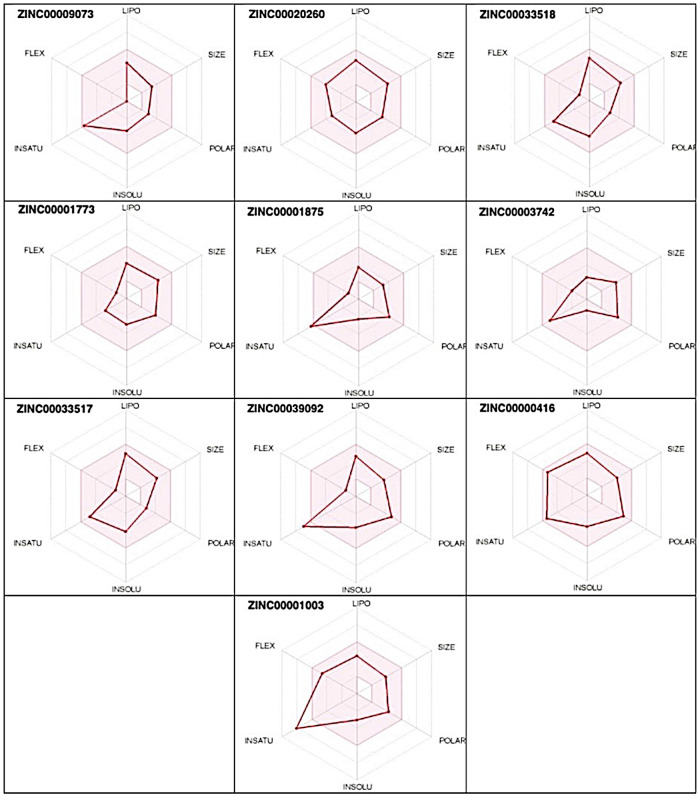
Radar charts of the 10 candidate molecules.

The bioavailability radars of the top 10 shortlisted compounds are shown in Fig 4. From the radar plot, it can be seen that ZINC00009073 (Apomorphine), ZINC00020260 (Oxyphencyclimine), ZINC00033518 ((S)-canadine), ZINC00001773 (Naltrexone), ZINC00003742 (Norfloxacin), ZINC00033517 ((R)-canadine) and ZINC000000416 (Labetalol) are likely to be orally bioavailable molecules as their physicochemical properties (red line radar) lie within the optimal zone (pink region). However, ZINC00001875 (Oxolinic acid), ZINC00039092 (Hesperetin) and ZINC00001003 (Benorilate) show low saturation (fraction of carbon sp^3^ (Fsp^3^) hybridization) values, i.e., 0.23, 0.19 and 0.12, respectively (S3 Table), making them not good for oral bioavailability. Additionally, from the *Brain Or IntestinaL EstimateD permeation* (BOILED-Egg) analysis (S1 Fig), the blue dots in egg white and egg yolk show Apomorphine, (S)-canadine, Naltrexone, Norfloxacin, Hesperetin, Labetalol and (R)-canadine as P-glycoprotein (P-gp) substrates while the red dots show that Oxolinic acid, Oxyphencyclimine and Benorilate have structural barriers that do not allow them to bind to P-gp causing problems in drug excretion triggering other toxicity outcomes [39].

### Stability evaluation of the proposed protein-ligand complexes

**Fig 5:**
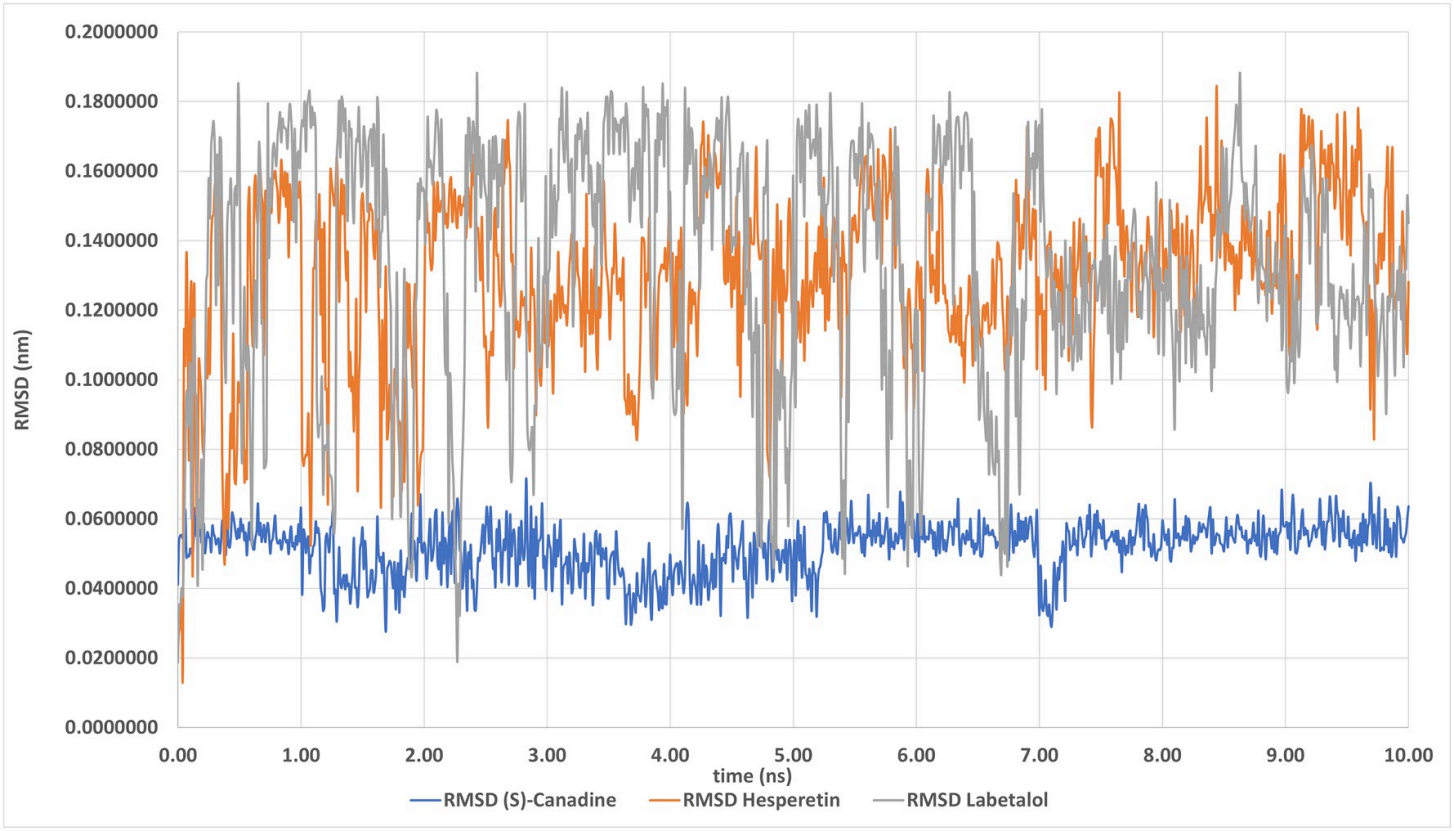
RMSD—Ligand heavy atoms in time of simulation.

(S)-canadine, hesperetin or labetalol formed stable protein-ligand complexes during molecular dynamics, remaining at the docking site for at least 10 ns by visual inspection in VMD. RMSD values for heavy atoms of ligands during 10 ns simulation for the three compounds were 0.05 ± 0.007; 0.13 ± 0.024; 0.13± 0.035 (mean ± SD), respectively (Fig 5), indicating very small changes in the ligand positions. (S)-canadine had a value of <0.1, which indicates the high stability of this complex. The (S)-canadine complex formed with this compound was the most constant in terms of time and molecular dynamics, having lowest RMSD (Fig 5). This compound could be further investigated for the development of new PCSK9 inhibitory drugs.

## Discussion

One of the major causes of atherosclerosis is the formation of arterial plaques, which eventually lead to arterial obstruction and heart attack. Furthermore, elevated levels of apoB-100 and LDL cholesterol are directly related to atherosclerotic cardiovascular disease. The lipid cycle generally starts with the secretion of immature VLDL from the liver. It also contains apoB-100, cholesteryl esters, triglycerides and cholesterol. During circulation in the blood, VLDL mature and become a source of energy in adipose tissues, skeletal and cardiac muscles [40]. The exchange and removal of triglycerides is one of the functions of VLDL, leading to the conversion of VLDL into intermediate-density lipoproteins (IDL) [41]. Some of these IDL can be destroyed by the liver during endocytosis, but IDL with a higher amount of cholesterol is converted to LDL, which also contains apoB-100. This apoB-100 binds to LDLR at its *N*-terminal domain and subsequently passes through via endocytosis in the acidic endosome. Ultimately, LDLR separates from LDL and is recycled to the cell membrane [42]. At acidic pH, conformational changes in LDLR can increase PCSK9 binding affinity towards LDLR in comparison to LDL [10]. Although mutations can destroy the catalytic activity of PCSK9, it does not interfere with its binding to LDLR. An interruption in the secretion of this protein may lead to inhibition of its binding to LDLR. PCSK9 mRNA degradation can eventually inhibit its association with LDLR or a small molecule designed against this can also inhibit such association [43]. Due to interaction of PCSK9 with LDLR (EGF-A domain or epidermal growth factor-like repeats), endocytosis of LDL is inhibited, which leads to atherosclerosis. To stop the accumulation of LDL, the interaction of PCSK9 to LDLR may be inhibited. PCSK9 is synthesized in the ER and transported to the plasma membrane to bind to LDLR. Many of the biological processes are regulated by protein-protein interactions. One of the promising and well-known drug discovery pipelines is to regulate these interactions. Therefore, to stop the EGF domain of LDLR from interacting with PCSK9, the target site where the interaction occurs needs to be defined.

It is known that anti-hypertensive drugs play an essential role in atherosclerosis, especially ACE inhibitors [44]. ACEi are potential inhibitors of plaque formation [45]. Experimental, epidemiological and clinical trials have shown that ACE inhibitors improve arterial endothelial function and hence slow down the progression of atherosclerosis [46]. They have been shown to reduce erythropoietin (EPO) levels and hematocrit. Several types of bioactive peptides can be regulated by ACEi such as angiotensin 1–7 and substance P. In hematopoiesis, involvement of these peptides has been observed. Erythropoiesis can be inhibited by decreasing the level of angiotensin II. ACEi also inhibit the renin-angiotensin system [47].

Various ACE inhibitors such as Captopril, Zofenopril, Enalapril, Ramipril, Quinapril, Perindopril, Lisinopril, Benazepril, Fosinopril, Cilazapril, Moexipril, Trandolapril, Allicin and Teprotide have been used for this research. Captopril is a compound that inhibits LDL oxidation, which is known to contribute to atherosclerosis progression [48]. Zofenopril and Enalapril are two ACEi that can decrease vascular damage and are involved in enhancing the circulation of endothelial progenitor cells. They are effective in preventing endothelial damage and the progression of atherosclerosis [49]. Another type of ACE inhibitor is Quinapril, which can reduce the adverse effects of Propofol on hemostasis [50]. Perindopril reduces the effects of oxidized LDLs in plasma and decreases C-reactive protein (CRP), fibrinogen, MCP-1 and PAI-1 [51]. Lisinopril is a synthetic inhibitor of the angiotensin-converting enzyme that does not bind to any serum proteins other than ACE [14]. In case of hypertension, combined use of amlodipine and an ACE inhibitor is beneficial. The combined use of amlodipine, a calcium-channel blocker, and benazepril, an ACE inhibitor, decrease arterial stiffness and ventricular hypertrophy [52]. Another most used drug for treating hypertension, Fosinopril, strongly binds ACE with an S-score of −18.9225 kcal·mol^-1^ [53]. Cilazapril (CIL) is a type of ACE inhibitor that can normalize blood pressure [54]. Another ACE inhibitor, Moexipril, can promote neuronal survival due to free radical scavenging [55]. Trandolapril and Ramipril are synthetic ACE inhibitor drugs that control high blood pressure and congestive heart failure. Allicin and Teprotide are natural compounds present in garlic, onion and snake venom respectively, which are also active ACEi [14].

ACE inhibitors (S1 Table) have proven to play an active role in the suppression of atherosclerosis, hence were incorporated in the drug discovery via pharmacophore modeling which was further screened against the ZINC database. Newly discovered compounds (1033) from the ZINC database were evaluated for their binding affinity using AutoDock tools. Various parameters like lower binding energy, interaction in the active site, and numbers of hydrogen bonds were computed using the software. Comparative analysis on these parameters outlined a group of top 10 molecules, which are the best drug candidates for atherosclerosis (S2 Table). It was found that some of the virtually screened molecules from the ZINC database gave good interaction(s), while few gave no interaction or interaction with less affinity. In the results, the top 10 compounds with binding energies from -9.8 to -8.2 kcal/mol (S2 Table), are comparable to the reference compounds (S1 Table) and hence are favorable and valid potential drug candidates for targeting PCSK9 in atherosclerosis. In other words, in this research, the binding affinity of the top selected molecules showed the best affinity with the PCSK9 target protein for atherosclerosis. In addition, Molinspiration, SWISSADME and admetSAR were used for computational structural analysis, drug-likeliness, ADME properties and oral bioavailability of these molecules.

The Molinspiration Cheminformatics website is helpful for the calculation of essential molecular properties, molecular processing, bioactivity and the high-quality illustration of molecules. The predicted Molinspiration enzyme and protease inhibitor bioactivity scores of many top ten compounds were <0, suggesting inhibitory properties. Ligand efficiencies (LE) for derivatives, calculated from docking energies and known number of heavy atoms, theoretical logP and logP/LE (LELP), divided by the number of binding sites were within the range of drug-likeliness (S4 Table). The values of the listed parameters were in similar ranges as the values for known anti-atherosclerotic medicines used as a starting point for our studies (S1 Table). If a molecule does not violate two or more of the Lipinski rules of five, it can be a potential orally active drug. The four key physicochemical parameters stated in Lipinski rule of five include molecular weight (MW ≤ 500 Da), lipophilicity (log P ≤ 5), hydrogen bond donors (HBDs; sum of NH and OH) ≤ 5, hydrogen bond acceptors (HBAs; Sum of N and O) ≤ 10. This rule describes pharmacokinetic drug properties, including absorption, distribution, metabolism and excretion (ADME) [56]. All the molecules were found to follow Lipinski, Veber and other rules (S3 and S4 Tables). A radar plot represents six physicochemical properties including lipophilicity (XLOGP3: between -0.7 and+5.0), size (MW:150-500g/mol), polarity (TPSA:20–130 Å^2^), solubility (INSOLUL: log S <6), saturation (INSTAU: sp3 hybridized carbon fraction >0.25 and <1.0) and flexibility (FLEX: rotatable bonds >0 and <9) that facilitate the overall drug development process. The pink zone depicts the optimal range of physicochemical properties for a drug to be orally bioavailable. If the radar of a molecule lies entirely in the pink zone (Fig 4), then it is likely to be an orally bioavailable drug [29, 57]. It was found in SWISSADME results (S3 Table) that all top 10 shortlisted compounds have a bioavailability score of 0.55 and are likely to be bioavailable.

Based on all these results, it can be concluded that the following compounds like (S)-canadine, hesperetin or labetalol, seem to be the most promising candidates for further research and possible design of new medicines based on their pharmacophore structure as they have heavy atoms, LE and LELP values in a range similar to known ACE inhibitors (S1 and S4 Tables). They were, therefore, chosen for further molecular dynamics studies of their complexes with PCSK9 based on AutoDock Vina results. All three compounds appeared to form stable PCSK9-ligand complexes remaining at the docking site for at least 10 ns during molecular dynamics and their RMSD values for heavy atoms were small. A considerable advantage of (S)-canadine, the alternative name (S)-Tetrahydroberberine, is its significantly lower RMSD (Fig 5) that helps to generate a very stable complex with PCSK9. This is an interesting result, because reports suggest that berberine exhibits PSCK9 inhibitory properties [37]. However, the main inhibitory mechanisms of berberine on PCSK9 are described as ‘the suppression of PCSK9 transcription’ [58] and that ‘berberine significantly increases LDLR expression through a posttranscriptional mechanism that stabilizes the LDLR mRNA, and decreases the expression and secretion of PCSK9 [37]. The direct action of such compounds as inhibitors is unknown and this research sheds some light on both (S)-canadine and berberine which have a very similar pharmacophore structure that differs mainly in the degree of saturation with hydrogen atoms. Berberine is an aromatic compound in contrast to (S)-canadine. An experimental study on mice found that berberine slows down the progression of atherosclerosis in apolipoprotein E knockout (ApoE-/-) cholesterol-fed mice [59]. Moreover, the network pathways analysis of Huanglian Jiedu Decoction, a traditional Chinese medicinal approach, has shown that (S)-canadine and berberine are two of the main ingredients that are likely to play an active role against atherosclerosis [60]. An *in vivo* study conducted on hesperetin to evaluate its role in LDLR expression proposed that hesperetin dosage (200μM) likely spurs the expression of LDLR gene which enhances mRNA level of transcription factors, SREBP-1 and SREBP-2, leading to decreased LDL-C level in plasma, hence, lowering the risk for CVD [61]. Studies also show that SREBP-2 activation leads to the co-expression of PCSK9 and LDLR which leads to degradation of LDLR, thus, increasing LDL-C circulation [62, 63]. So, the effect of hesperetin is likely to be cancelled.

Studies found that as the Fsp^3^ value increases, the saturation increases [64, 65]. The relationship of Fsp^3^ with the solubility and melting point properties of a dataset of 1000 compounds revealed a positive correlation between Fsp^3^ and solubility whereas, a negative correlation with melting point [64]. Chemical entities with lower melting points display better absorption in comparison to compounds with higher melting points [66]. Therefore, low saturation (Fsp^3^) of a chemical entity leads to a high melting point that might limit the drug absorption, ultimately affecting the oral bioavailability. Thus, we hypothesize that hesperetin displays a low saturation value (S3 Table) making it less likely to be orally bioavailable. Various studies show that hesperetin is a poor orally bioavailable compound [67, 68], but it can be improved by certain chemical techniques [69], thus, proving our results. (S)-canadine and (R)-canadine have distinctive bioavailability properties as these two molecules are permeable to the blood brain barrier (BBB) and can reach their target easily (S1 Fig and S3 Table). These compounds also show high gastrointestinal tract absorption and the ability to bind to P-gp making them good orally bioavailable drug candidates [70]. Therefore, based on our current studies, (S)-canadine pharmacophore structure may act as an inhibitor for the PCSK9 protein molecule.

Pharmacophore as a drug discovery approach was applied to detect novel inhibitors for PCSK9 using known ACE inhibitors. The literature was surveyed to find out the best structure for PCSK9 and its active site identified for known ACE inhibitors. The molecular docking results highlighted the role of drug discovery in atherosclerosis by incorporating computational and unbiased methods. In this research, the binding affinity was investigated and the top 10 molecules showing the best affinity with the target for atherosclerosis included ZINC00009073, ZINC00020260, ZINC00033518, ZINC00001773, ZINC00001875, ZINC00003742, ZINC00033517, ZINC00039092, ZINC00000416 and ZINC00001003. These top drug candidates were analyzed and passed the Lipinski rule of 5 test. Several compounds from the virtual screening, and back search of drug databases, were found to be plant-derived compounds like (S)-canadine, hesperetin or identified as the antihypertensive drug–labetalol. These compounds formed stable protein-ligand complexes as shown by molecular dynamics. (S)-canadine deserves special attention, because the complex formed with this compound was the most unchanged in the time of molecular dynamics, with the lowest RMSD, and can be further investigated for the development of new PCSK9 inhibitory drugs.

## Supporting information

S1 Fig BOILED-Egg prediction of gastrointestinal absorption and blood-brain barrier permeability for top 10 compounds. (PDF)

S1 TableKnown ACE inhibitors.Their SMILES codes and docking results with the target (PDB ID– 6U26), MW–predicted molecular weight, HA–the number of heavy atoms, milogP − Molinspiration logP, EI − Enzyme inhibitor Molinspiration bioactivity score v2018.03, PI–Protease inhibitor Molinspiration bioactivity score v2014.03, LE–Ligand Efficiency–($-RTln\frac{K_{d}}{HA}$ or $-\frac{\Delta G^{0}}{HA}$), where *ΔG*^*0*^ is predicted as the standard free energy of ligand binding, LELP=milogPLE.(PDF)

S2 Table Molecular docking results for top 10 molecules/drug candidates. (PDF)

S3 Table Parameters such as molecular formula/weight, atoms, fractions, hydrogen bond acceptors and donors for the top 10 compounds. (XLSX)

S4 TableZINC, PubChem, Molinspiration examination and Lipinski rule of 5 for top 10 molecules.MW–predicted molecular weight (gmol^-1^/Da), HA–the number of heavy atoms, milogP–Molinspiration logP, EI and PI–Enzyme and Protease inhibitor Molinspiration bioactivity scores v2014.03, respectively (significant values bold), LE=-RTlnKd=-ΔG0HA, LELP=miLogPLE, A*log*P–Octanol/water partition coefficient for hydrophobicity analysis, HBA–hydrogen bond acceptors, HBD–hydrogen bond donors, RotB–rotatable bonds.(PDF)
