# Supplementary material for: Finding inhibitors for PCSK9 using computational methods
Source: PLoS One. 2021 Aug 5;16(8):e0255523. doi: 10.1371/journal.pone.0255523 (PMC8341581; doi:10.1371/journal.pone.0255523)
Supplement: S1 Fig — (PDF) [file pone.0255523.s001.pdf]

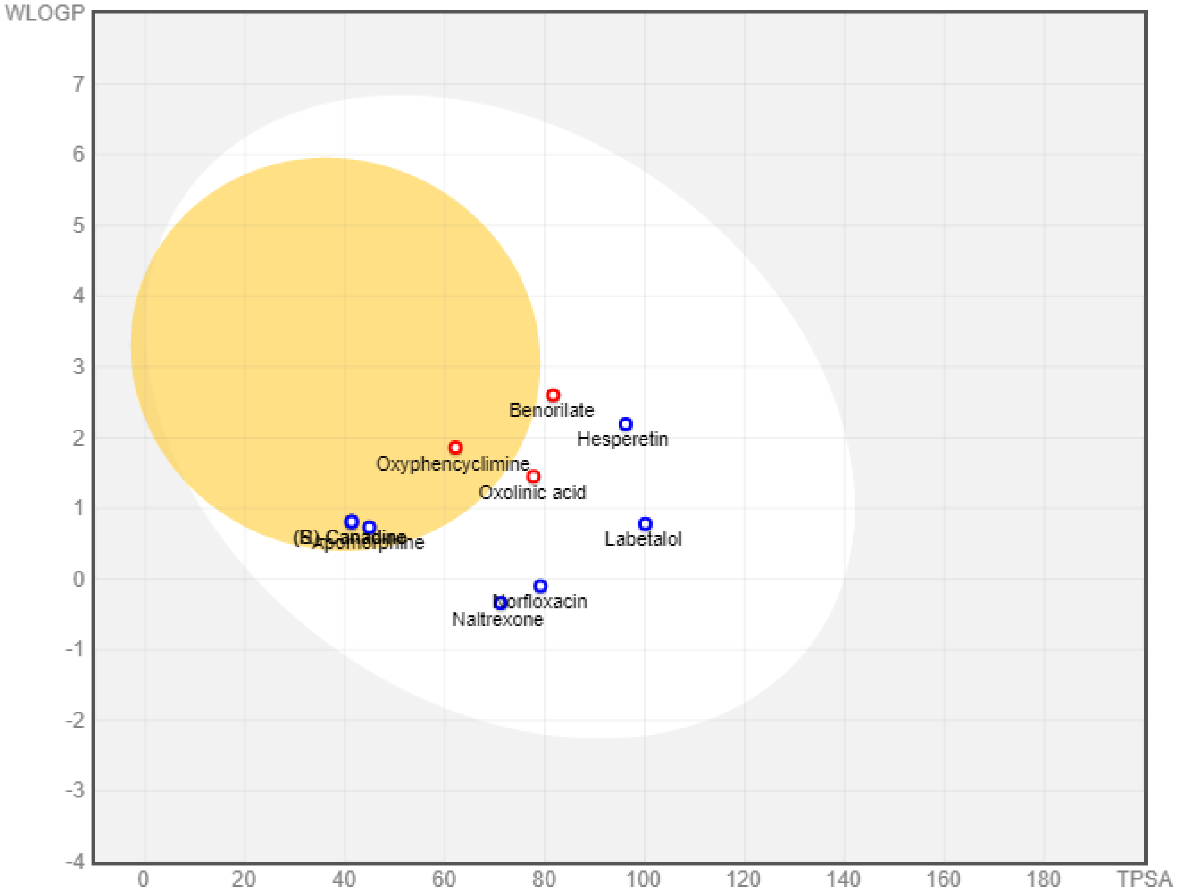

Fig. 1. Boiled egg prediction of gastrointestinal absorption and blood brain barrier permeability for top 10 compounds
