# Supplementary material for: Finding inhibitors for PCSK9 using computational methods
Source: PLoS One. 2021 Aug 5;16(8):e0255523. doi: 10.1371/journal.pone.0255523 (PMC8341581; doi:10.1371/journal.pone.0255523)
Supplement: S1 Table — Their SMILES codes and docking results with the target (PDB ID– 6U26), MW–predicted molecular weight, HA–the number of heavy atoms, milogP − Molinspiration logP, EI − Enzyme inhibitor Molinspiration bioactivity score v2018.03, PI–Protease inhibitor Molinspiration bioactivity score v2014.03, LE–Ligand Efficiency–(-RTlnKdHA or -ΔG0HA), where ΔG0 is predicted as the standard free energy of ligand binding, LELP=milogPLE. (PDF) [file pone.0255523.s002.pdf]

S1 Table. Known ACE inhibitors. Their SMILES codes and docking results with the target (PDB ID – 6U26), MW – predicted molecular weight, HA – the number of heavy atoms, milogP − Molinspiration logP, EI − Enzyme inhibitor Molinspiration bioactivity score v2018.03, PI – Protease inhibitor Molinspiration bioactivity score v2014.03, LE – Ligand Efficiency – ($-RTln\frac{K_d}{HA}$ or $-\frac{\Delta G^0}{HA}$), where ΔG^0 is predicted as the standard free energy of ligand binding, $LELP = {milogP}/{LE}$

Inhibitors Image	CANONICAL SMILES	Molecule Name/PubChem ID	Residues	Binding Energy (kcal·mol^{-1})	MW (Da)	HA	milogP	EI	PI	LE<br>LELP
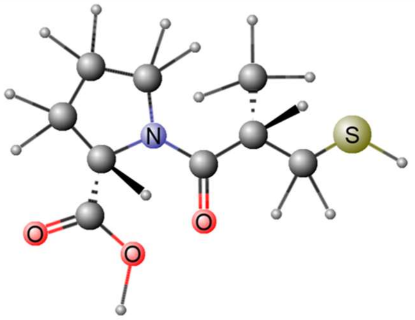	CC(CS)C(=O)N1CCCC1C(=O)O	Captopril/ 44093	ASP 651<br>ARG 525	-6.0	217.29	14	-1.09	**0.50**	**0.97**	0.4<br>-2.5
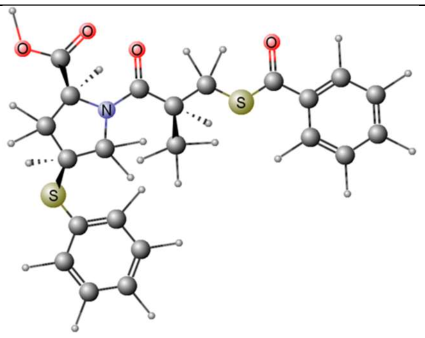	CC(CSC(=O)C1=CC=CC=C1)C(=O)N2CC(CC2C(=O)O)SC3=CC=CC=C3	Zofenopril/92400	ARG 458<br>TRP 461	-8.3	429.56	29	2.36	0.14	**0.79**	0.3<br>8.2
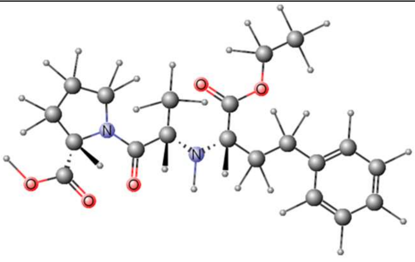	CCOC(=O)C(CCC1=CC=CC=C1)NC(C)C(=O)N2CCCC2C(=O)O	Enalapril/ 5388962	ARG 458	-7.8	376.45	27	0.51	0.18	**0.70**	0.3<br>1.8

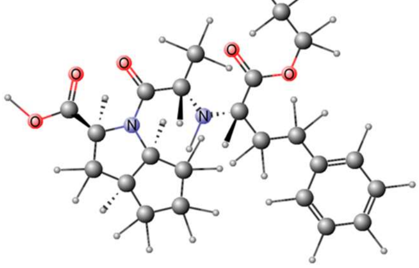	CCOC(=O)C(CCC1=CC=CC=C1)NC(C)C(=O)N2C3CCCC3CC2C(=O)O	Ramipril/5362129	ARG 458<br>ARG 458	-8.4	416.52	30	2.40	**0.23**	**0.78**	0.3<br>8.6
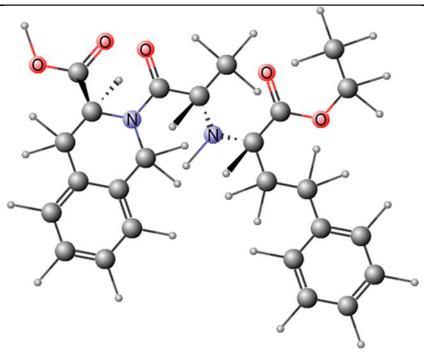	CCOC(=O)C(CCC1=CC=CC=C1)NC(C)C(=O)N2CC3=CC=CC=C3CC2C(=O)O	Quinapril/54892	ARG 458	-8.9	438.52	32	1.49	0.14	**0.50**	0.4<br>5.4
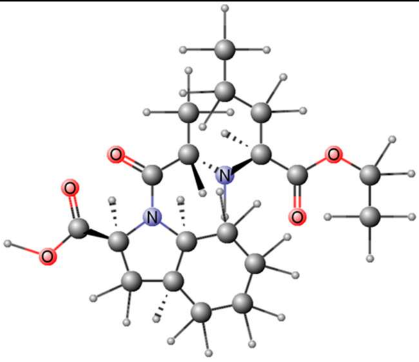	CCCC(C(=O)OCC)NC(C)C(=O)N1C2CCCCC2CC1C(=O)O	Perindopril/107807	ARG 458	-7.2	368.47	26	2.02	0.20	**0.83**	0.2<br>8.3
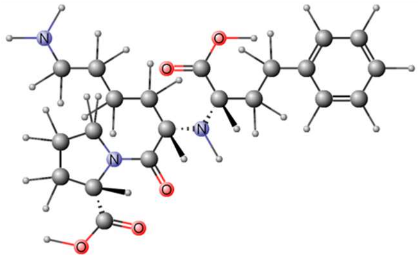	C1CC(N(C1)C(=O)C(CCCCN)NC(CCC2=CC=CC=C2)C(=O)O)C(=O)O	Lisinopril/5362119	ARG 458	-7.8	405.50	29	-2.44	0.39	**0.91**	0.3<br>2.2

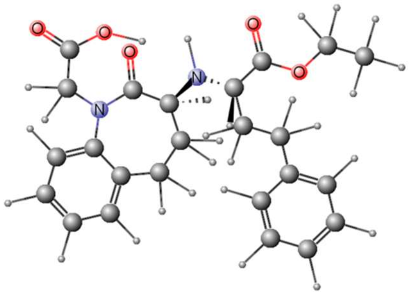	CCOC(=O)C(CCC1=CC=CC=C1)NC2CCC3=CC=CC=C3N(C2=O)CC(=O)O	Benazepril/5362124	ARG 458<br>ARG 476	-7.5	424.50	31	2.52	0.10	0.43	0.2<br>10.4
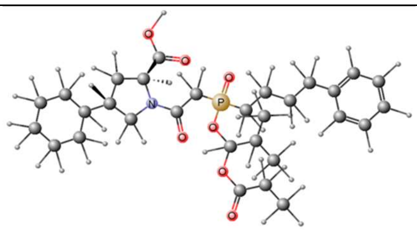	CCC(=O)OC(C(C)C)OP(=O)(CCCCC1=CC=CC=C1)CC(=O)N2CC(CC2C(=O)O)C3CCCCC3	Fosinopril/55891	ARG 458<br>ARG 357<br>ALA463	-7.3	563.67	39	4.59	**0.41**	**1.03**	0.2<br>23.9
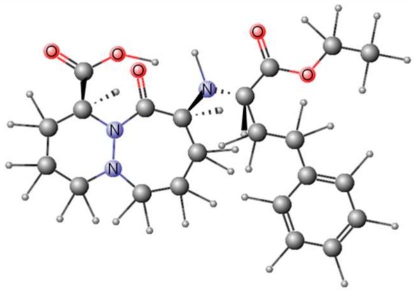	CCOC(=O)C(CCC1=CC=CC=C1)NC2CCCN3CCCC(N3C2=O)C(=O)O	Cilazapril/56330	ARG 357	-7.7	417.51	30	0.57	0.07	**0.64**	0.3<br>2.2
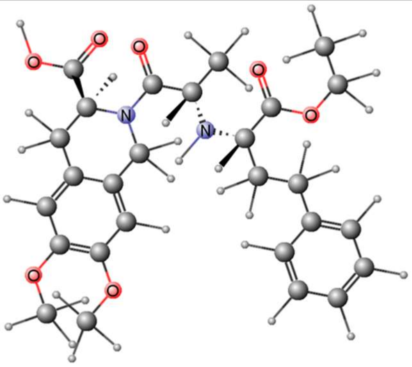	CCOC(=O)C(CCC1=CC=CC=C1)NC(C)C(=O)N2CC3=CC(=C(C=C3CC2C(=O)O)OC)OC	Moexipril/91270	ARG 458	-8.6	498.58	36	1.12	0.09	**0.38**	0.2<br>4.7

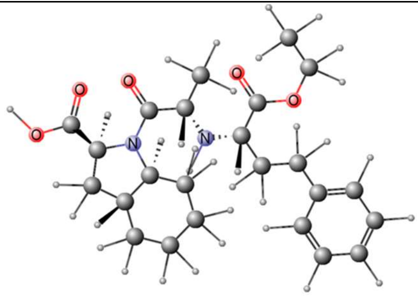	CCOC(=O)[C@H](CCC1=CC=CC=C1)N[C@@H](C)C(=O)N2[C@H]3CCCC[C@@H]3C[C@H]2C(=O)O	Trandolapril/5484727	ARG 458	-8.4	430.55	31	2.90	0.17	**0.76**	9.3<br>10.7
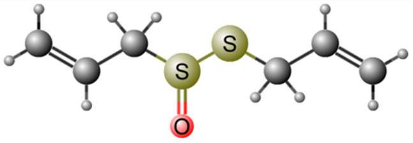	C=CCSS(=O)CC=C	Allicin/65036	ARG 458	-8.1	162.28	9	2.06	-<br>1.52	-<br>1.40	0.9<br>2.3
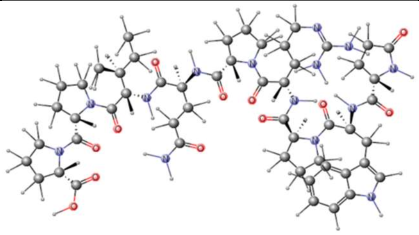	CCC(C)C(C(=O)N1CCCC1C(=O)N2CCCC2C(=O)O)NC(=O)C(CCC(=O)N)NC(=O)C3CCCN3C(=O)C(CCCN=C(N)N)NC(=O)C4CCCN4C(=O)C(CC5=CNC6=CC=CC=C65)NC(=O)C7CCC(=O)N7	Teprotide/443376	ARG 458<br>ARG 357<br>ASN298<br>CYS323<br>ARG 458<br>TRP 461	-9.8	1101.28	79	-4.66	-<br>3.77	-<br>3.68	0.1<br>-7.6
