# Supplementary material for: Finding inhibitors for PCSK9 using computational methods
Source: PLoS One. 2021 Aug 5;16(8):e0255523. doi: 10.1371/journal.pone.0255523 (PMC8341581; doi:10.1371/journal.pone.0255523)
Supplement: S2 Table — (PDF) [file pone.0255523.s003.pdf]

S2 Table. Molecular docking results for top 10 molecules/drug candidates.

ZINC ID/IDENTIFIED NAME/SMILES	BINDING ENERGY (kcal·mol^{-1})	INTERACTIONS(S)	MOLECULAR INTERACTION(S)
			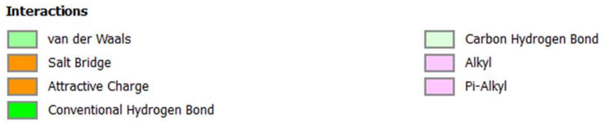
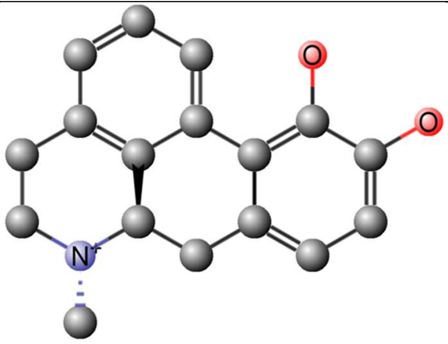 ZINC00009073/Apomorphine/C[N@@H+]1CCC2=C3[C@H]1CC1=CC=C(O)C(O)=C1C3=CC=C2	-9.8	ARG357 O -->O20	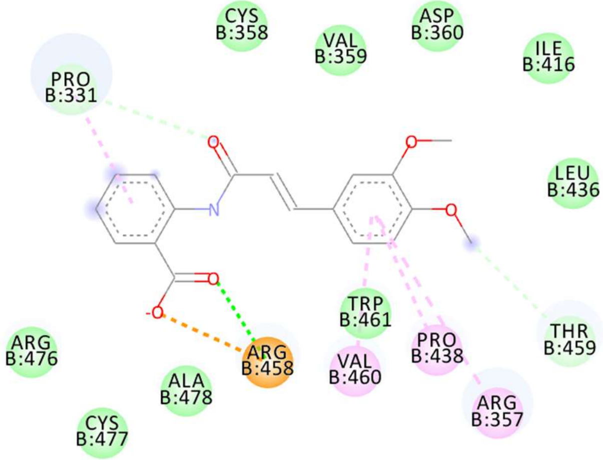
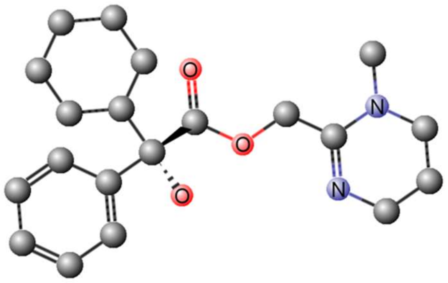 ZINC00020260/Oxyphencyclimine/CN1CCCN=C1COC(=O)[C@](O)(C1CCCCC1)C1=CC=CC=C1	-9	ARG458 HH12 --> O23	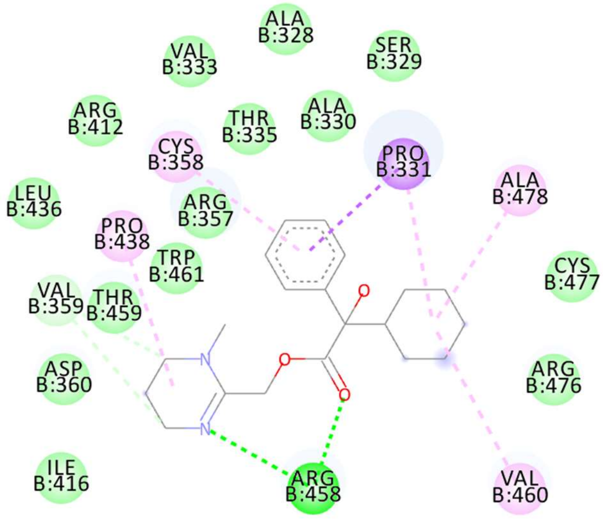

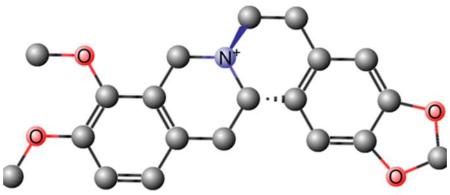<br>ZINC00033518/(S)-Canadine/COC1=C(OC)C2=C(C[C@@H]3[N@H+](CCC4=C3C=C3OCOC3=C4)C2)C=C1	-9	VAL359 O --> O25	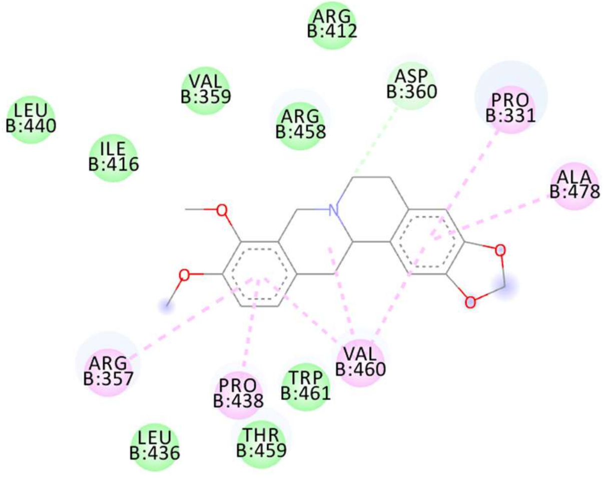
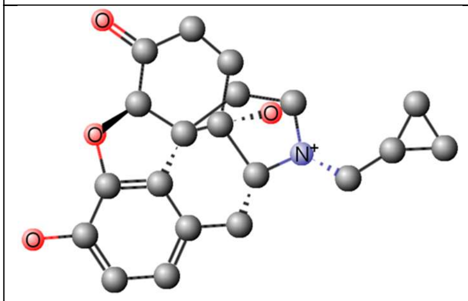<br>ZINC00001773/Naltrexone/OC1=CC=C2C[C@H]3[N@@H+](CC4CC4)CC[C@@]45[C@@H](OC1=C24)C(=O)CC[C@@]35O	-8.8	ARG458 HH22 --> O25 & ARG458 HH12 --> O25	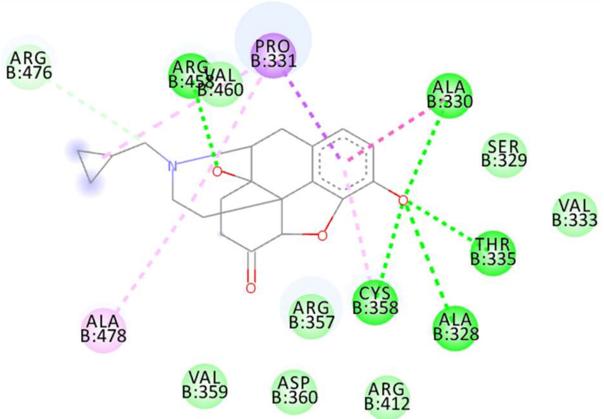
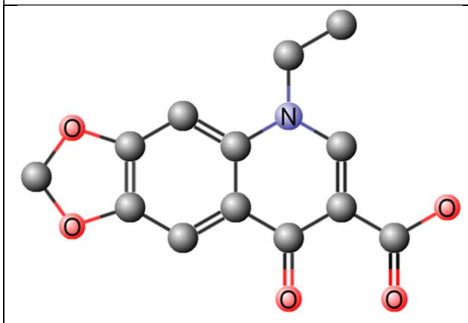<br>ZINC00001875/Oxolinic acid/CCN1C=C(C(O)=O)C(=O)C2=CC3=C(OCO3)C=C12	-8.6	TRP 461 HE1 --> O15	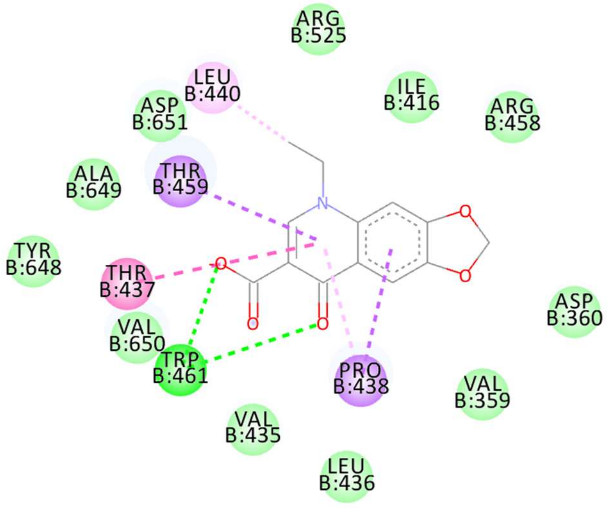

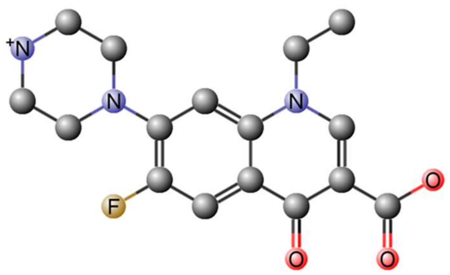<br>ZINC00003742/Norfloxacin/CCN1C=C(C(O)=O)C(=O)C2=CC(F)=C(C=C12)N1CC[NH2+]CC1	-8.6	ARG458 HH12 --> O22	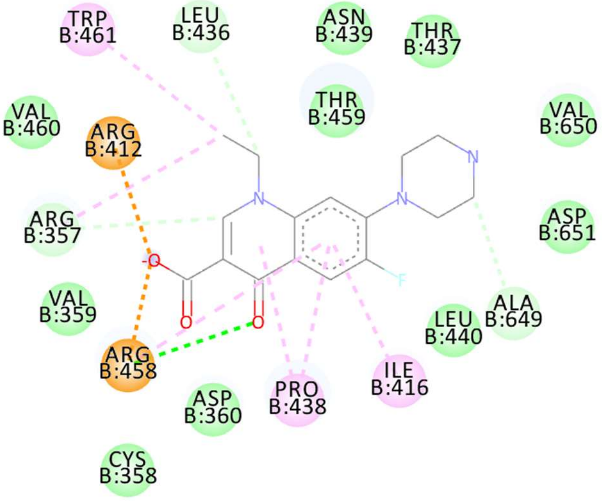
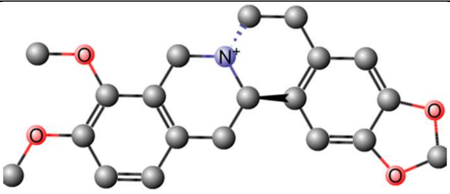<br>ZINC00033517/(R)-Canadine/COC1=C(OC)C2=C(C[C@H]3[N@@H+](CCC4=C3C=C3OCOC3=C4)C2)C=C1	-8.6	ARG458 HH12 --> O22	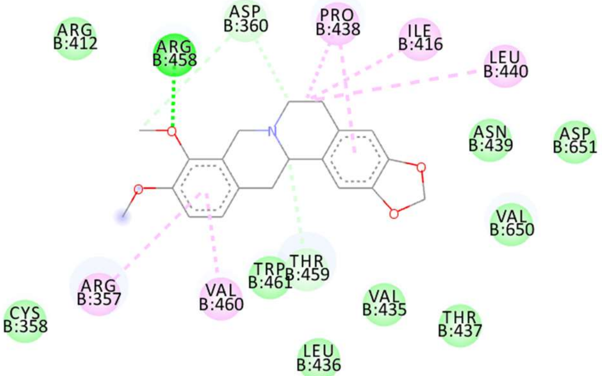
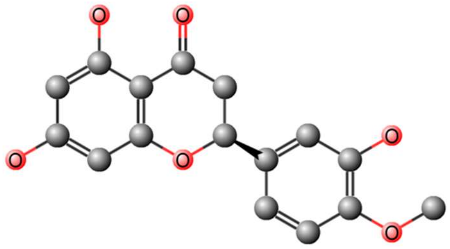<br>ZINC00039092/Hesperetin/COC1=C(O)C=C(C=C1)[C@@H]1CC(=O)C2=C(O1)C=C(O)C=C2O	-8.4	TRP461 HN --> O19	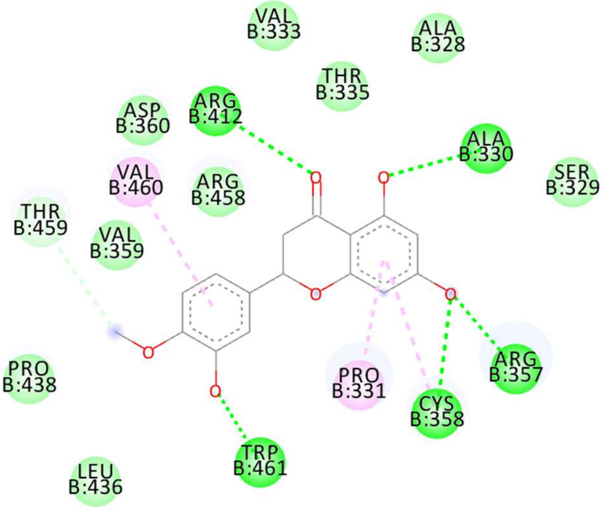

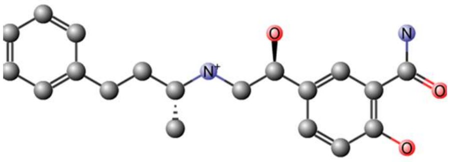<br>ZINC00000416/Labetalol/C[C@H](CC C1=CC=CC=C1)[NH2+]C[C@@H](O) C1=CC(C(N)=O)=C(O)C=C1	-8.3	TRP461 HE1 --> O22	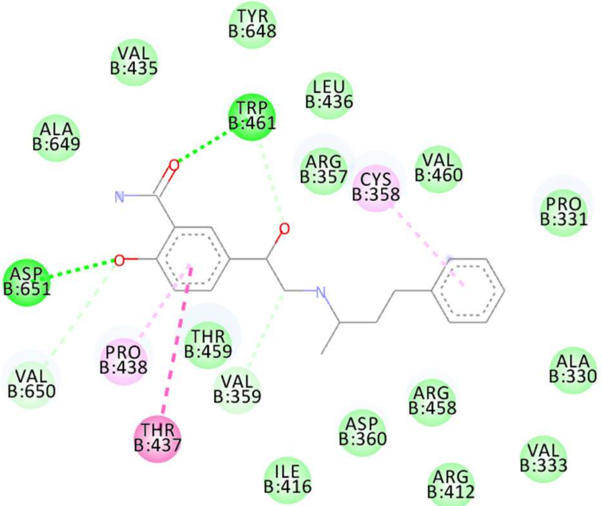
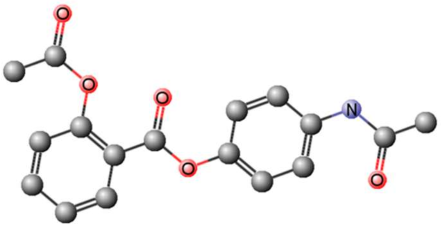<br>ZINC00001003/Benorilate/CC(=O)NC1 =CC=C(OC(=O)C2=C(OC(C)=O)C=CC =C2)C=C1	-8.2	ARG458 HH12 --> O22	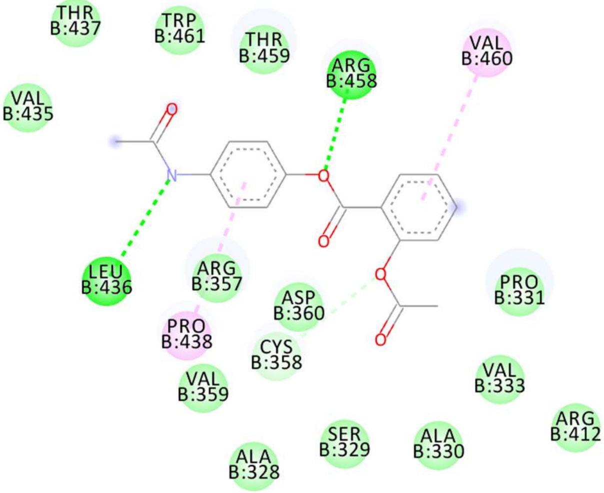
