# Supplementary material for: Finding inhibitors for PCSK9 using computational methods
Source: PLoS One. 2021 Aug 5;16(8):e0255523. doi: 10.1371/journal.pone.0255523 (PMC8341581; doi:10.1371/journal.pone.0255523)
Supplement: S4 Table — MW–predicted molecular weight (gmol-1/Da), HA–the number of heavy atoms, milogP–Molinspiration logP, EI and PI–Enzyme and Protease inhibitor Molinspiration bioactivity scores v2014.03, respectively (significant values bold), LE=-RTlnKd=-ΔG0HA, LELP=miLogPLE, AlogP–Octanol/water partition coefficient for hydrophobicity analysis, HBA–hydrogen bond acceptors, HBD–hydrogen bond donors, RotB–rotatable bonds. (PDF) [file pone.0255523.s005.pdf]

S4 Table. ZINC, PubChem, Molinspiration examination and Lipinski rule of 5 for top 10 molecules. MW – predicted molecular weight (gmol^{-1}/Da), HA – the number of heavy atoms, milogP – Molinspiration logP, EI and PI – Enzyme and Protease inhibitor Molinspiration bioactivity scores v2014.03, respectively (significant values bold), $LE = -RTlnK_d = -{}^{\Delta G^0}/_{HA}$, $LELP = {}^{miLogP}/_{LE}$, AlogP – Octanol/water partition coefficient for hydrophobicity analysis, HBA – hydrogen bond acceptors, HBD – hydrogen bond donors, RotB – rotatable bonds.

**ZINC ID, Identified names, PubChem CIDs, Molinspiration SMILES, Molinspirarition chemical formula**	**~ MW (gmol^{-1}/Da)**	**HA**	**milogP**	**EI**	**PI**	**LE LELP**	**AlogP**	**HBA**	**HBD**	**RotB**	**LIPINSKI RULE OF 5 using KNIME**
ZINC00009073, Apomorphine, 6931310 (6005), c1cc2c3c(ccc(c3O)O)C[C@@H]3c2c(c1)CCN3C 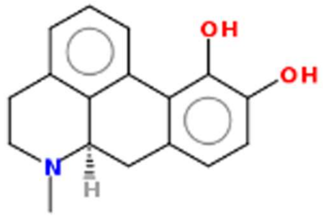	268.34/327.34	24	3.16	-0.09	-0.25	0.4 7.7	1.43	2	3	0	**PASS**
ZINC00020260, (1-Methyl-5,6-dihydro-4H-pyrimidin-2-yl)methyl (2S)-2-cyclohexyl-2-hydroxy-2-phenylacetate, Oxyphencyclimine, 667690 (4642), [C@@](O)(C1CCCCC1)(c1ccccc1)C(=O)OCC1=NCCCN1C	344.46/344.45	25	3.57	**0.32**	0.20	0.4 9.9	2.73	5	1	5	**PASS**

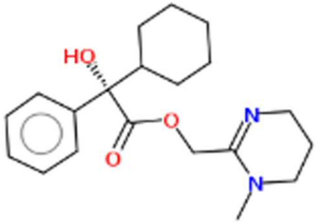											
ZINC00033518, (S)-Canadine, 21171 (6919570),<br>c1cc(c(c2c1C[C@H]1c3cc4c(cc3CCN1C2)OCO4)OC)OC<br>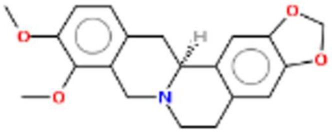	340.40/339.39	25	2.99	-<br>0.06	-<br>0.26	0.4<br>8.3	1.67	4	1	2	**PASS**
ZINC00001773, Naltrexone, 5360515 (23253567),<br>c1cc(c2c3c1C[C@@H]1[C@@]4(CCC(=O)[C@@H]([C@]34CCN1CC1CC1)O2)O)<br>O<br>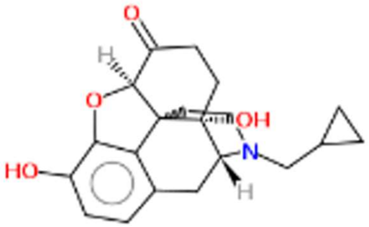	342.42/341.41	25	1.37	**0.48**	**0.30**	0.4<br>3.9	0.11	4	3	2	**PASS**

ZINC00001875, Oxolinic acid, 4628, c1c2c(cc3c1OCO3)n(cc(c2=O)C(=O)O)CC<br>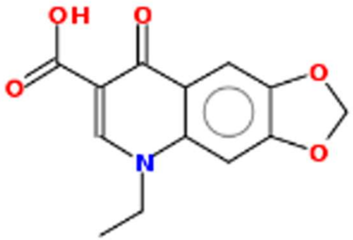	261.23/261.23	19	0.68	**0.21**	-<br>0.64	0.5<br>1.5	1.42	5	1	2	**PASS**
ZINC00003742, Norfloxacin, 4539,<br>c1c2c(cc(c1F)N1CCNCC1)n(cc(c2=O)C(=O)O)CC<br>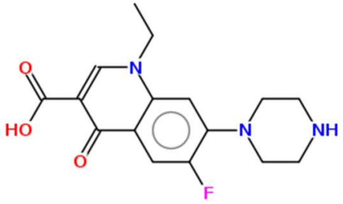	320.34/319.34	23	-0.69	0.17	-<br>0.27	0.4<br>-1.8	0.24	4	2	3	**PASS**
ZINC00033517, (R)-Canadine, 443422 (6919571),<br>c1cc(c(c2c1C[C@@H]1c3cc4c(cc3CCN1C2)OCO4)OC)OC<br>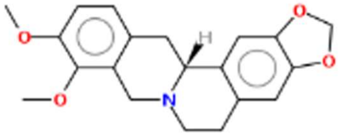	340.40/339.39	**25**	2.99	-<br>0.06	-<br>0.26	0.3<br>8.7	1.67	4	1	2	**PASS**

ZINC00039092, Hesperetin, 72281,<br>c1c2c(c(cc1O)O)C(=O)C[C@H](O2)c1ccc(c(c1)O)OC<br>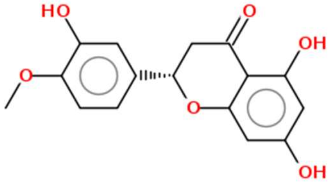	302.28/302.28	22	1.94	0.16	-<br>0.13	0.4<br>5.1	2.52	6	3	2	**PASS**
ZINC00000416, 2-Hydroxy-5-[(1S)-1-hydroxy-2-[[(2R)-4-phenylbutan-2-yl]amino]ethyl]benzamide Labetalol, 134045 (25271683), C([C@@H](O)c1ccc(O)c(c1)C(=O)N)N[C@H](C)CCc1ccccc1<br>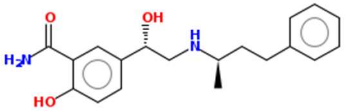	329.42/328.41	24	2.85	0.16	**0.30**	0.3<br>8.2	1.11	3	4	8	**PASS**
ZINC00001003, Benorilate, 21102, C(=O)(c1ccccc1OC(=O)C)Oc1ccc(cc1)NC(=O)C<br>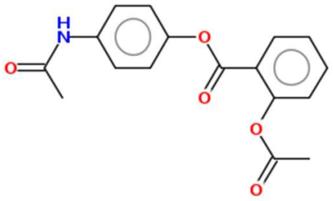	313.31/313.31	23	2.61	-<br>0.24	-<br>0.26			**0.4**<br>**7.3**	2.79	4	**PASS**
